# Landscape Pattern Changes in the Xingkai Lake Area, Northeast China

**DOI:** 10.3390/ijerph16203820

**Published:** 2019-10-10

**Authors:** Xiaohui Liu, Yuan Zhang, Guihua Dong, Guanglei Hou, Ming Jiang

**Affiliations:** 1 Key Laboratory of Wetland Ecology and Environment, Northeast Institute of Geography and Agroecology (IGA), Chinese Academy of Sciences, Changchun 130102, China; liuxh@iga.ac.cn (X.L.); jiangm@iga.ac.cn (M.J.); 2 Key Laboratory of Geographic Information Science (Ministry of Education), School of Geographic Sciences, East China Normal University, Shanghai 200241, China; 3 China National Environmental Monitoring Center (CNEMC), Beijing 100012, China; donggh@cnemc.cn; 4 Northeast Institute of Geography and Agroecology (IGA), Chinese Academy of Sciences, Changchun 130102, China; houguanglei@iga.ac.cn; 5 Jillin Provincial Joint Laboratory of Changbai Mountain Wetland and Ecology, Northeast Institute of Geography and Agroecology (IGA), Chinese Academy of Sciences, Changchun 130102, China

**Keywords:** spatiotemporal variations of landscape pattern, landscape fragmentation, landscape stability, land use changes, the Xingkai Lake area

## Abstract

Understanding landscape change is important for ecologically sustainable development. In this paper, we assessed the spatiotemporal variations of landscape pattern in the Xingkai Lake area using remote sensing data from 1982, 1995, 2000, 2005, 2010, and 2015. Landscape patterns of marshlands, paddy fields, dry farmlands, and their combinations were analyzed at class and landscape levels. We examined the stability of landscape types through principal component analysis based on class level indices for landscape types. The results indicated that marshland areas decreased significantly by 33.87% but paddy fields increased by 1.84 times from 1982 to 2015. The largest conversion of dry farmlands to paddy fields was 90.88 km^2^ during the period 2010–2015. In contrast, the largest conversion of paddy fields to dry farmlands was 86.03 km^2^ during the period 2000–2005. The difference in relative change revealed that dry farmlands had experienced a greater relative change than paddy fields since 2000. The interspersion and juxtaposition index decreased, while the number of patches grew. This showed that landscape fragmentation was increasing and the landscape pattern was becoming dispersed. Marshlands were more stable than paddy fields and dry farmlands across all time periods, except for the year 2005.

## 1. Introduction

The increasing exploitation of natural resources has led to an excessive depletion of resources and has changed the environment [1]. This unfolding ecological crisis can directly affect landscape pattern changes [2]. The rapid transformation in land use from land development directly affects landscape patterns [3,4]. The interaction between land use change and landscape pattern change is a focus of environmental change research because of the rapid land transformation [5]. The landscape pattern and its changes reflect the combined influence of natural and human systems [6,7].

Wetland ecosystems have experienced the most rapid decline among different ecosystems in the world [8]. Agricultural expansion has caused half of the world’s wetlands to be lost over the past century, and population growth had put additional pressure on wetlands [9,10]. Wetland loss and degradation has affected the well-being of many local communities [11,12,13]. However, effective restoration is often hindered by limited information on the historical process of landscape change. Therefore, key features of the spatial and temporal variations of landscape patterns among marshlands, paddy fields, and dry farmlands are still uncertain.

Landscape ecology deals with the patterning of ecosystems in space [14]. Landscape patterns indicate the actual spatial composition of landscape elements. Landscape pattern change is the most intuitive reflection of land use changes [15]. In the early 1950s, descriptive research on landscape patterns was carried out by Forman, and quantitative research in the field began in the 1970s [16,17]. Landscape stability is an important part of landscape ecology research, considered as a landscape that has been stable (the tendency of a perturbed system to return toward an undisturbed state) and which will not undergo tremendous structural changes in the short term [18,19]. This also means the natural processes that contribute to the functions and sustainability will not be disrupted. However, when analyzing landscape stability, it is difficult to make a quantitative analysis of landscape stability only considering landscape heterogeneity, diversity, and landscape pattern. As such, it was assessed quantitatively in this study. The landscape pattern index (LPI) is commonly used in landscape pattern research. The LPI is a quantitative index that can condense landscape pattern information and reflect landscape structural compositions and spatial allocation. Several landscape pattern indices have been developed to evaluate landscape stability, and principal component analysis is often used to construct a model that can accurately reflect landscape stability. The spatial arrangement of a landscape also has a decisive influence on landscape stability in space [20].

The conflicts between wetland conservation and cropland development are increasingly prominent, but less attention has been paid to the relationships between marshlands, paddy fields, and dry farmlands. It is useful to investigate the spatial and temporal dynamics of these landscape classes, especially over recent years. Further, the influence of landscape dynamics on landscape stability needs to be determined. Remote sensing and geographic information system technologies are often used to analyze land use changes. We aimed to analyze the transformation of marshlands into paddy fields or dry farmlands, or the relationships between paddy fields and dry farmlands along a time series in our study. The Xingkai Lake area, Northeast China, acted as the case study area. The area has typical natural marshlands and a history of reclamation to support a nationally important grain commodity base.

This study is focused on the landscape pattern changes experienced in the Xingkai lake area over the past thirty years. Land use changes around Xiangkai Lake in 1982, 1995, 2000, 2005, 2010, and 2015 were quantified by remote sensing data analysis. The spatiotemporal variations of landscape pattern at class and landscape levels were revealed by landscape pattern indices in Fragstats software. Based on the landscape pattern indices, we analyzed landscape stability using principal components analysis. Our findings are applicable for the effective planning and management of land resources.

## 2. Materials and Methods

### 2.1. Study Area

The Xingkai Lake area (45°01′–47°34′N, 131°58′–133°07′E) covers 2.59 × 10^3^ km^2^ within the Sanjiang Plain, Northeast China. This region lies in a temperate monsoon climate zone [21]. The annual mean temperature is 3 °C, with an average temperature of −18 °C in January and 21 °C in July. The annual mean precipitation is 654 mm. Precipitation is concentrated in summer, accounting for about 70% of the mean annual precipitation. The mean annual evaporation is 1450 mm [22,23].

Our study area contains seven types of land use: marshland, paddy field, dry farmland, forestland, grassland, residence, and lake. The vegetation in the marshland area is mainly *Deyeuxia angustifolia* and *Carex* plants. The main species of crops in the Xingkai Lake area are soybean, corn, and rice.

The implementation of China’s agricultural modernization policy since 1978 and rapid socioeconomic development has led to marshland reclamation in the Sanjiang Plain [24,25], resulting in the exploitation of marshlands and development of croplands in this region. Based on the history of reclamation of wetlands, three land use types, namely, marshland, paddy field, and dry farmland, were analyzed in our study.

### 2.2. Data Sources

The land use data in 1982 were derived from the Institute of Remote Sensing and Geographic Information Research Center of the Northeast Institute of Geography and Agroecology (http://marsh.neigae.csdb.cn/). Data for the other five periods, namely 1995, 2000, 2005, 2010, and 2015, were derived from Landsat images (http://glovis.usgs.gov/) covering this region at a resolution of 30 m. The phases of images were selected from June to October, as this was convenient for discrimination of land use type characteristics [26,27]. Using ArcGIS10.2.1 software, we obtained the information about land cover categories using supervised classification based on the Landsat TM432 band composite images. Information was acquired regarding spatiotemporal distribution and different land use types. From this, we analyzed the landscape pattern changes combined with landscape pattern indices.

### 2.3. Relative Land Use Change

We define the relative land use change in Equation (1), which expresses the land use changes for different periods from 1982 to 2015. The positive and negative values show whether the landscape area is expanding or decreasing.

(1) $$R_{S}=\frac{U_{f}-U_{i}}{U_{i}}\times \frac{1}{T}\times 100\%$$

where *R_S_* is relative land use change, *U_i_* and *U_f_* are land use types at the beginning and end stages, and *T* is time interval. 

### 2.4. Landscape Pattern and Stability

Class- and landscape-level indices were used to characterize the landscape pattern changes in this study. Landscape pattern indices help to determine digital information about landscape composition, the dynamics of landscape patterns, and the spatial configuration among landscape types. Indices were calculated by Fragstats version 4.2.1 software. The indices were NP, LPI, FRAC_AM, COHESION, SPLIT, and AI for the class level, and CONTAG, IJI, SHDI, SHEI, NP, and COHESION for the landscape level. Descriptions for these indices are outlined in Table 1.

We established the model for evaluating landscape type stability using principal component analysis [28]. The six indices at the class level were used to assess landscape type stability. We constructed a standard matrix and then gained the eigenvalue and contribution ratios from principal component analysis to assess relative importance using PASW (Predictive Analytics Software) Statistics 18 software. We also obtained the load matrix and correlation coefficient matrix from principle component analysis. The weight of the principal component and landscape type stability can be calculated using the following equations.

(2) $$W_{i}=\frac{\lambda_{i}}{\sum_{i=1}^{n}\lambda_{i}}$$

where *W_i_* is weight of the *i*th principal component and *λ_i_* is eigenvalue of the *i*th principal component.

(3) $$F_{i}=a_{i}X_{1}+b_{i}X_{2}+c_{i}X_{3}+d_{i}X_{4}+e_{i}X_{5}+f_{i}X_{6}$$

where *F_i_* is the *i*th principal component; *X_i_* is an index at the class level (*i* = 6); and *a*_i_, *b*_i_, *c*_i_, *d*_i_, *e*_i_, and *f*_i_ are correlation coefficient matrices for the *i*th principle component.

(4) $$F=\overset{n}{\underset{i=1}{\sum }}W_{i}F_{i}$$

where *F* is the grading score for landscape type stability, *W_i_* is the weight of the *i*th principal component, and *F_i_* is the *i*th principal component. 

## 3. Results

### 3.1. Land Use Changes

**Figure 1:**
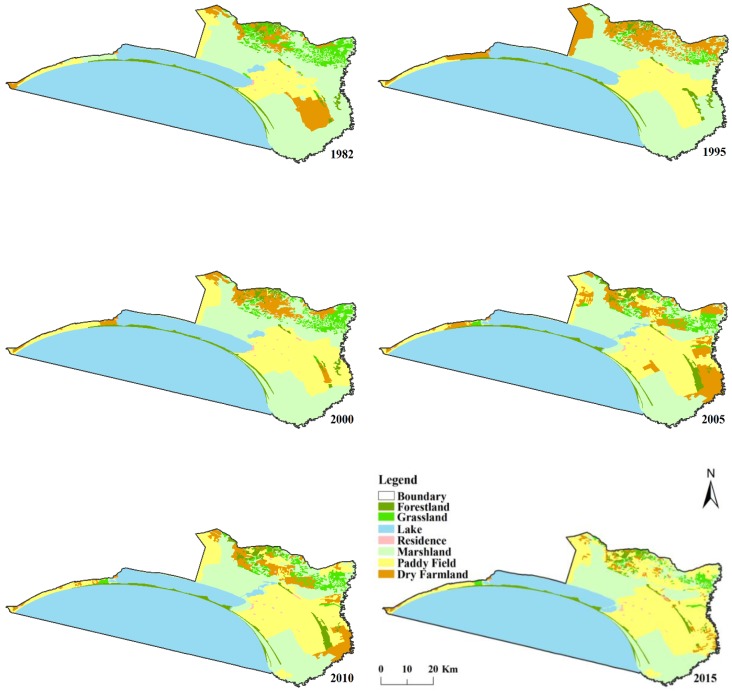
Spatial distribution of land use in the Xiangkai Lake area from 1982 to 2015.

We measured the spatial distribution and area ratios of marshlands, paddy fields, and dry farmlands for the years 1982, 1995, 2000, 2005, 2010, and 2015. Marshlands occupied 787.2 km^2^ (30.42%) of the total study area in 1982 (Figure 1).

Our analysis showed that the marshlands decreased by 33.87% and dry farmlands decreased by 64.72% from 1982 to 2015, but paddy fields increased by 1.84 times during this period (Table 2). The ratio of paddy fields and dry farmlands together (25.71%) has exceeded that of marshlands (20.58%) since 2005. The ratio of paddy fields increased much more than marshlands decreased from 1982 to 2015.

**Figure 2:**
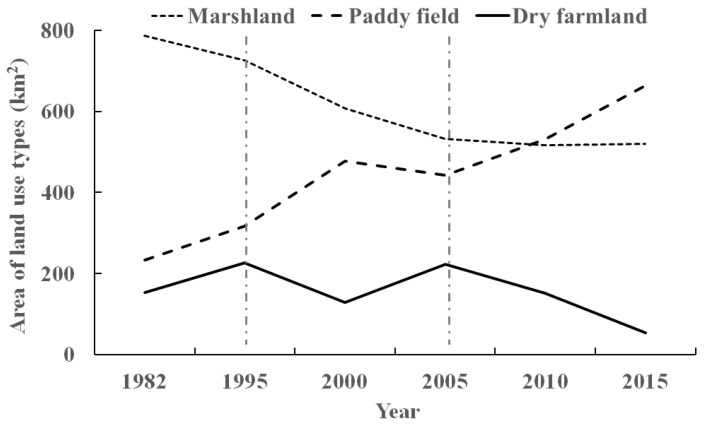
Changes of land use over different years.

The relative changes in marshlands and dry farmlands was negative during 1995–2000 and 2005–2010, but that of paddy fields was always positive (Table 3). Trends for marshlands, paddy fields, and dry farmlands for different years can be divided into three stages (Figure 2). Firstly, before 1995, the original marshlands declined because of increasing land reclamation. Secondly, from 1995 to 2005, the marshland area was reduced sharply, but the paddy fields and dry farmlands fluctuated, with both tending toward the opposite trend. Thirdly, after 2005, the marshlands area was relatively stable, while paddy fields and dry farmlands showed opposite trends. The total paddy fields area reached a maximum in 2015. The area of dry farmlands converted to paddy fields was 76.22 km^2^ during 2005–2010 and 90.88 km^2^ during 2010–2015 (Table 3), which were the highest values across the different periods.

The two conversion processes of dry farmlands to paddy fields and of marshlands to paddy fields were very significant in every time period. The largest conversion of paddy fields to dry farmlands was 86.03 km^2^ for the period 2000–2005, while the largest conversion of dry farmlands to paddy fields was 90.88 km^2^ for the period 2010–2015 (Table 3). The relative change of dry farmlands was –12.86%, but for paddy fields it was 4.96%, so there was a bigger amplitude of dry farmland changes between 2010 and 2015. The conversion of marshlands to paddy fields was significantly higher than that of marshlands to dry farmlands across the different periods, except 2000–2005. Compared with the relative change of paddy fields, the changing range of dry farmlands was greater than that of paddy fields after 2000. This might be influenced by the initial conversion of dry farmlands to paddy fields in the late 1990s.

As shown in Table 3, marshland conversion to paddy fields totaled 253.24km^2^ for the period 1982–2015, with a relative change of 5.58%. In particular, the area of marshlands converted to paddy fields occupied 32.17% of the total marshland area in 1982. The area of dry farmlands converted to paddy fields was 82.01% of the total dry farmland area in 1982.

### 3.2. Landscape Pattern Changes

The Δ means the difference of indices between 1982 and 2015. The ΔLPI and ΔFRAC_AM values for marshlands were both higher than those for paddy fields and dry farmlands for the period 1982–2015 in Table 4, which showed greater human disturbance and severe landscape fragmentation. In terms of ΔNP, values for paddy fields and dry farmlands were significantly higher than for marshlands, which indicated that the development intensity of paddy fields and dry farmlands had increased, especially after 1995. Dry farmlands were greatly dispersed compared with the marshlands and paddy fields according to the ΔSPLIT, which was the highest. The ΔAI of marshlands was the smallest at 0.2237%, while the ΔCOHESION of dry farmlands was the largest at up to 1.4776, so the patch connectivity in the landscape was not compact between 1982 and 2015.

We obtained a standard matrix for NP, LPI, FRAC_AM, COHENSION, SPLIT, and AI, as shown in Table 5. The two principal components were extracted and the eigenvalues were 4.598 and 1.402, respectively. From this we produced a correlation coefficient matrix (Table 6 and Table 7).

Expressions of two principal components are listed according to Equation (3) (Table 7). The first principal component (*F*_1_) had remarkable higher loads for LPI, FRAC_AM, COHESION, and SPLIT. The second principal component (*F*_2_) had high loads for NP and AI. Using Equations (2) and (4), landscape type stability could be expressed. The grading values for landscape type stability are shown in Table 8.

$$F_{1\;}=0.1354X_{1}+0.2168X_{2}+0.1965X_{3}+0.2076X_{4}-0.2143X_{5}+0.1567X_{6}\;F_{2\;}=0.5580X_{1}+0.0562X_{2}+0.3060X_{3}-0.2132X_{4}+0.1219X_{5}-0.4946X_{6}\;F=76.63\%F_{1}+23.37\%F_{2}$$

The marshland area ratio was higher (30.42%) in 1982 than the other two landscape types (paddy fields and dry farmlands). The highest LPI (93.9018) and SPLIT (1.1333) values were for marshlands in 1982, which had the highest score (0.89) for relatively stable land type in 1982. However, the maximum difference for marshlands was 0.50 and the stability of marshlands varied over different years. Marshlands were more stable than paddy fields and dry farmlands across all years examined, except for 2005.

Landscape-level indices for the three landscape types together (marshlands, paddy fields, and dry farmlands) are analyzed in Table 9. At the landscape level, clear evidence of the fragmentation process was observed (Table 9). IJI significantly decreased (5.3509%), implying a more dispersed landscape pattern from 1982 to 2015. Meanwhile, NP rapidly increased by 1.7167 times, which led to a clear fragmentation process.

CONTAG showed several differences with a range of 0.3074%, and there were more fragmented patches because our CONTAG was concentrated at 50% (a range of 0–100). COHESION was about 99.8, with no particularly obvious change over different years, which showed that landscape connectivity had been sustained.

SHDI and SHEI were both at their maximum in 2005, which meant that the landscape tended to become more heterogeneous over time and there was a more even distribution of the patch types in the landscape.

## 4. Discussion

The study presented the changes in landscape pattern over different time periods in the Xingkai Lake area. The time period with the largest land use conversion was found. The study revealed that landscape fragmentation was further aggravated until 2015. Human disturbances are the important reason for landscape fragmentation [29]. The building of artificial canals for paddy field cultivation and the increase in canal densities has led to a decrease of plant community diversity in the wetlands of the Sanjiang Plain. The natural and seminatural areas have been gradually replaced by artificial and semiartificial areas [6,30]. These human activities have influenced the water circulation and the landscape pattern. Reclamation has put great pressure on marshlands in the Xingkai Lake area. This mirrors human disturbance to peatlands, where approximately 15% globally and over 50% of peatlands in Europe have been drained for agricultural use [31].

In our study, landscape pattern indices were selected from the literature and combined with our own understanding of landscape patterns. This means that there was some subjectivity in the selected process. Our selection might not necessarily show the complete relationship between landscape type stability and landscape pattern indices. We used principal component analysis to evaluate landscape type stability based on landscape pattern indices. Notably, landscape pattern indices focus on calculating the geometric relationship between patch types, but do not involve the measurement of biomass or species diversity within patches. Therefore, the results cannot fully reflect the characteristics of landscape stability. There are two main considerations in this paper, as follows. Landscape pattern indices can be used to quantitatively reflect the spatial distribution characteristics of the landscape, and on this basis, principal component analysis could be used to reveal the landscape type stability. However, the spatial scale is very important for landscape stability analysis [17]. Further research should be undertaken to improve the accuracy of ecosystem stability classification criteria or construct a new ecosystem stability index system. In other words, a more innovative approach should be proposed for studying ecosystem stability and the landscape pattern indices influencing it.

The landscape pattern was obviously fragmented in our study over the past thirty years. It was characterized by a sharp increase of NP and decrease of IJI. Our results provide support for studying landscape pattern changes at a class level. Therefore, it was a remarkable increase in scale from the previous study [32]. Marshlands were more dispersed and had poor patch connectivity, and marshland stability declined. These findings are consistent with the conclusions of other related studies [28,33,34].

Compared to the loss of abundant resources, the loss and degradation of limited resources has an even stronger impact on human well-being [13]. To protect natural wetland ecosystems and landscape stability, ecological compensation pilot studies have been launched since 2014 in China [35]. The scope of compensation mainly includes internationally important wetlands or national natural reserves and their surrounding areas along the migration routes of waterfowl (Ministry of Finance, Ministry of Agriculture (2014) 9). Xingkai Lake National Natural Reserve is the first ecological compensation pilot in China and the largest waterfowl migration stopover in Northeast Asia. The numbers of wild ducks and goose occupied 70%–90% of the total number of water birds, and the ecological compensation ranged from 19.78 × 10^3^ to 27.91 × 10^3^ yuan per hectare [36]. The willingness to protect natural ecosystems should be improved with the increasing public recognition of nonmarket service values [37,38,39]. Wetlands in the Xingkai Lake area and other areas may not be restored to their original state by depending only on restoration programs [36,40]. Human interventions related to biodiversity have great impacts on wetland ecosystems. Preventing or reversing these influences should be the main direction for restoration efforts. China proposes to establish a natural protected area system, which would be mainly composed of national parks. The first national park on earth was established in 1872. In 2016, China’s first national park, Sanjiangyuan National Park, marked the first step for this country.

## 5. Conclusions

This study revealed the time periods with the largest land use conversions, namely for marshland conversion to paddy fields or dry farmlands, during the period 1982–2015. We also quantitatively identified the key landscape pattern indices that reflected the landscape changes in this region. Landscape fragmentation increased until 2015. The marshlands were the most stable land use type, followed by paddy fields and dry farmlands. Investigating the dynamics of wetland landscape patterns can explain changes in wetland landscapes over time, as well as provide theoretical support for wetland resource use, conservation, and management.

## Figures and Tables

**Table 1:** Class- and landscape-level indices.

Indices	Description	Units	Metrics
Number of patches (NP)	Patch numbers	None	[1,∞)
Largest patch index (LPI)	Percentage of the maximum patch area to total patch area	Percent	(0,100]
Area-weighted mean fractal dimension index (FRAC_AM)	Spatial shape complexity of patches or landscape	None	[1,2]
Patch cohesion index–(COHESION)	Physical connection with the corresponding patch type	None	(0,100)
Splitting index (SPLIT)	Dispersion of spatial distribution	None	[0,100]
Aggregation index (AI)	Degree of aggregation of spatial patterns	Percent	[0,100]
Contagion index (CONTAG)	Degree of agglomeration or extension of different patch types in the landscape	Percent	(0,100]
Interspersion and juxtaposition index (IJI)	Interspersion or juxtaposition among patch types in the landscape	Percent	(0,100]
Shannon’s diversity index (SHDI)	Landscape heterogeneity	None	[0,∞)
Shannon’s evenness index (SHEI)	Even distribution of patch types in the landscape	None	[0,1]

**Table 2:** Percentages of land use types from 1982 to 2015 (%).

Land Use Types	1982	1995	2000	2005	2010	2015	Rate of Change
Marshland	30.42	28.03	23.58	20.58	19.94	20.12	−33.87
Paddy Field	9.03	12.28	18.44	17.12	20.55	25.65	184.18
Dry Farmland	5.93	8.73	4.99	8.59	5.86	2.09	−64.72

**Table 3:** Conversion and relative change of land use over different periods.

Conversion and Relative Change	Types	1982–1995	1995–2000	2000–2005	2005–2010	2010–2015	1982–2015
Land use conversion area (km^2^)	Conversion of Paddy Field to Dry Farmland	43.28	2.09	86.03	0.04	7.37	2.75
	Conversion of Dry Farmland to Paddy Field	71.42	66.12	33.48	76.22	90.88	125.80
	Conversion of Marshland to Paddy Field	49.35	101.57	29.21	10.85	22.64	253.24
	Conversion of Marshland to Dry Farmland	40.57	13.51	62.53	5.64	2.66	23.92
Relative land use change (%)	Marshland	−0.60	−3.23	−2.48	−0.62	0.18	−1.03
	Paddy Field	2.77	10.03	−1.43	4.02	4.96	5.58
	Dry Farmland	3.64	−8.57	14.39	−6.35	−12.86	−1.96

**Table 4:** Class level indices for landscape types in the Xingkai Lake area.

Year	Type	*NP*	*LPI*	*FRAC_AM*	*COHESION*	*SPLIT*	*AI*
1982	Marshland	67	93.9018	1.2047	99.9618	1.1333	98.9530
	Paddy field	5	72.3448	1.1192	99.8753	1.7784	99.2230
	Dry farmland	25	52.5981	1.1048	99.4726	3.2739	98.3227
1995	Marshland	78	48.0251	1.1701	99.9055	2.1746	98.9122
	Paddy field	2	91.9215	1.1024	99.9477	1.1744	99.5758
	Dry farmland	42	29.7709	1.1501	99.6572	5.0290	97.7673
2000	Marshland	56	61.6955	1.1626	99.8482	2.0703	98.4998
	Paddy field	10	80.2958	1.1169	99.9256	1.5064	99.4703
	Dry farmland	21	42.5284	1.1570	99.6543	4.1157	97.7213
2005	Marshland	51	48.8452	1.1569	99.8456	2.5217	98.8124
	Paddy field	17	77.0674	1.1357	99.8564	1.6604	99.0833
	Dry farmland	46	31.1281	1.1270	99.4552	7.0326	97.9041
2010	Marshland	52	48.4528	1.1654	99.8497	2.5492	98.7085
	Paddy field	15	72.1722	1.1296	99.8595	1.8581	99.1776
	Dry farmland	34	32.6651	1.1477	99.4533	5.8030	97.4356
2015	Marshland	82	45.1822	1.1422	99.7694	2.7120	98.7293
	Paddy field	40	62.6888	1.1497	99.8480	2.3639	98.9454
	Dry farmland	81	10.1690	1.1189	97.9950	24.9259	94.1959
1982–2015	Marshland	15	−48.7196	−0.0625	−0.1924	1.5787	−0.2237
	Paddy field	35	−9.6560	0.0305	−0.0273	0.5855	−0.2776
	Dry farmland	56	−42.4291	0.0141	−1.4776	21.6520	−4.1268

**Table 5:** The standard matrix for landscape type stability indices.

Year	Type	X_1_	X_2_	X_3_	X_4_	X_5_	X_6_
1982	Marshland	1.0955	1.0143	1.1444	0.7350	−0.8456	0.2600
	Paddy field	−0.8638	−0.0292	−0.4389	0.4037	−0.2581	0.8444
	Dry farmland	−0.2318	−0.9851	−0.7055	−1.1388	1.1038	−1.1043
1995	Marshland	0.9820	−0.2676	0.8405	0.4377	−0.3090	0.1754
	Paddy field	−1.0171	1.1066	−1.1060	0.7065	−0.8090	0.9007
	Dry farmland	0.0351	−0.8390	0.2655	−1.1442	1.1180	−1.0761
2000	Marshland	1.1240	0.0100	0.6860	0.2779	−0.3597	−0.0730
	Paddy field	−0.7910	0.9950	−1.1474	0.8317	−0.7704	1.0345
	Dry farmland	−0.3330	−1.0050	0.4614	−1.1096	1.1301	−0.9615
2005	Marshland	0.7082	−0.1511	1.1076	0.5536	−0.4216	0.3440
	Paddy field	−1.1439	1.0670	−0.2709	0.6008	−0.7201	0.7826
	Dry farmland	0.4358	−0.9158	−0.8366	−1.1544	1.1418	−1.1266
2010	Marshland	0.9909	−0.1330	0.9963	0.5561	−0.4055	0.2973
	Paddy field	−1.0089	1.0598	−1.0037	0.5984	−0.7336	0.8177
	Dry farmland	0.0180	−0.9269	0.0075	−1.1544	1.1391	−1.1149
2015	Marshland	0.5981	0.2182	0.3279	0.5394	−0.5638	0.5366
	Paddy field	−1.1545	0.8729	0.7949	0.6145	−0.5908	0.6172
	Dry farmland	0.5564	−1.0911	−1.1228	−1.1539	1.1546	−1.1538

**Table 6:** The eigenvalue and contribution ratios for principal components.

Original Eigenvalue				Cumulative Loads of Extracted Factors		
Factors	Eigenvalue	Contribution ratio (%)	Cumulative contribution ratio (%)	Eigenvalue	Contribution ratio (%)	Cumulative contribution ratio (%)
1	4.598	76.626	76.626	4.598	76.626	76.626
2	1.402	23.374	100.000	1.402	23.374	100.000
3	1.89 × 10^−16^	3.14 × 10^−15^	100.000			
4	5.27 × 10^−17^	8.79 × 10^−16^	100.000			
5	−5.83 × 10^−17^	−9.72 × 10^−16^	100.000			
6	−2.53 × 10^−16^	−4.21 × 10^−15^	100.000			

**Table 7:** The load matrix and correlation coefficient matrix for principal component analysis.

Principal Component				
Load Matrix			Correlation Coefficient Matrix	
	1	2	1	2
X_1_	0.6226	0.7825	0.1354	0.5580
X_2_	0.9969	0.0788	0.2168	0.0562
X_3_	0.9032	0.4291	0.1965	0.3060
X_4_	0.9543	−0.2989	0.2076	−0.2132
X_5_	−0.9853	0.1710	−0.2143	0.1219
X_6_	0.7203	−0.6936	0.1567	−0.4946

**Table 8:** The grading scores for landscape type stability.

Year	Marshland	Paddy Field	Dry Farmland
1982	0.89	−0.22	−0.67
1995	0.46	−0.09	−0.36
2000	0.49	−0.06	−0.43
2005	0.50	0.03	0.53
2010	0.55	−0.10	−0.45
2015	0.39	0.21	−0.60

**Table 9:** Landscape-level indices for the three landscape types together in the Xingkai Lake area.

Year	*NP*	*CONTAG*	*IJI*	*COHESION*	*SHDI*	*SHEI*
1982	60	59.6894	89.3436	99.9186	0.8552	0.7785
1995	99	53.4747	51.7514	99.8400	0.9737	0.8863
2000	54	53.7783	89.4419	99.8092	0.9827	0.8945
2005	139	50.5749	97.8366	99.7933	1.0408	0.9474
2010	83	53.1673	99.1201	99.8033	0.9849	0.8965
2015	163	59.3820	83.9927	99.7890	0.8354	0.7604
1982–2015	103	−0.3074	−5.3509	−0.1296	−0.0198	−0.0181
